# The economic burden of CIDP in the United States: A case-control study

**DOI:** 10.1371/journal.pone.0206205

**Published:** 2018-10-23

**Authors:** Victoria Divino, Rajiv Mallick, Mitch DeKoven, Girishanthy Krishnarajah

**Affiliations:** 1 Health Economics and Outcomes Research, IQVIA, Fairfax, VA, United States of America; 2 Global Health Economics and Reimbursement Strategy, CSL Behring, King of Prussia, PA, United States of America; University of Paris XII, FRANCE

## Abstract

**Background:**

Chronic inflammatory demyelinating polyneuropathy (CIDP) is a rare neurological disorder of the peripheral nervous system. The economic burden of CIDP is not well understood.

**Objectives:**

To assess the economic and clinical burden of CIDP and to compare the incremental burden relative to a matched control group without CIDP.

**Methods:**

This retrospective case-control analysis was conducted using data from the IQVIA Real-World Data Adjudicated Claims. Adults newly diagnosed with CIDP between 7/1/2010 and 6/30/2014 were identified and direct matched to controls without CIDP. Baseline characteristics were assessed and compared over a 6-month pre-index period. Healthcare resource use, costs and clinical characteristics were assessed and compared over a 2-year follow-up. Total cost differences over the 2-year follow-up were compared between matched cohorts using a generalized estimating equation model.

**Results:**

The final sample comprised a total of 790 cases matched to 790 controls. Over the 2-year follow-up, cases more frequently experienced neuropathic pain, back pain and osteoarthritis and more commonly utilized opioids, anti-convulsants and anti-depressants. Compared to controls, more cases had ≥1 hospitalization (26.2% vs. 9.0%), and cases had a higher mean number of outpatient prescription fills (62.8 vs. 32.0) and physician office visits (34.7 vs. 13.0) (all p<0.0001). Cases had 7.5x higher mean total costs ($116,330 vs. $15,586, p<0.0001). Important cost drivers were costs for outpatient ancillary, radiology and HCPCS drugs (mean $76,366 vs. $4,292) and costs for inpatient care (mean $16,357 vs. $2,862) (both p<0.0001). Among cases, CIDP therapy (inclusive of both outpatient pharmacy and medical claims) accounted for 51.2% of mean total costs. After further adjusting for baseline clinical characteristics, cases were associated with a 6.1x increase in total costs compared to controls (p<0.0001).

**Conclusions:**

Our findings suggest a substantial clinical and economic burden among patients with CIDP relative to matched controls over a 2-year follow-up.

## Introduction

Chronic inflammatory demyelinating polyneuropathy (CIDP) is a rare, chronic, acquired immune mediated disorder of the peripheral nervous system, characterized by progressive, symmetrical limb weakness, large fiber sensory loss and loss of reflexes [1–3]. Initial symptoms include sensory loss, usually beginning in the legs, and patients may report impaired ambulation (e.g., difficulty walking, climbing stairs) [3]. In the United States (US), the annual incidence of CIDP has been estimated at 1.6 per 100,000 people while the prevalence has been estimated at 8.9 per 100,000 people [4].

There has been increasing concern about misdiagnosis and over-diagnosis of CIDP in current clinical practice [5,6]. Not surprisingly, in view of the potential for misdiagnosis, stringent electro-diagnostic tests are required for the diagnosis of CIDP, along with the assessment of clinical and laboratory features [7]. Several US payers mandate positive electrophysiological findings on at least three of four tests (per guidelines from the American Academy of Neurology [AAN]) as part of medical necessity for CIDP treatment, particularly intravenous immunoglobulin (IVIg) [1,8,9]. Furthermore, a differential diagnosis is necessary to exclude disorders that may similarly present as CIDP, such as other peripheral nerve disorders [5]. Timely and appropriate initiation of therapy early on in the course of disease is critical to prevent permanent disability [10].

The primary goals of treatment for CIDP are to reduce symptoms, improve functional status (e.g., reduce disability and handicap) and maintain long-term remission as possible [3]. According to joint guidelines on the management of CIDP from the European Federation of Neurological Societies and the Peripheral Nerve Society, IVIg (level A recommendation) or corticosteroids (level C recommendation) should be considered for induction of treatment in patients with CIDP that present with disabling symptoms [7]. A report of the AAN has stated that IVIg is effective and should be offered in the long-term treatment of CIDP (level A); however, data are insufficient to address the comparative efficacy of other CIDP treatments (e.g., steroids) [11]. Other treatment options for CIDP include plasma exchange (PE) and the addition of immunosuppressant or immunomodulatory drugs (e.g., azathioprine, mycophenolate motefil, rituximab) [3].

The economic burden of CIDP is not well understood. Two published studies have identified substantial costs associated with CIDP [12,13]. No published studies have evaluated the incremental burden of CIDP. Given the limited real-world data on the economic burden of CIDP, the objectives of this analysis were to assess the economic burden of CIDP in the 2 years following diagnosis, and to quantify the incremental burden of CIDP relative to a control group without CIDP, from the payer perspective, using a large, nationally representative database in the US. Secondary objectives were to assess patient characteristics and the clinical burden of CIDP.

## Methods

### Study overview

This retrospective case-control analysis was conducted using IQVIA Real-World Data (RWD) Adjudicated Claims, using data from January 2010 through June 2016.

### Data source

IQVIA RWD Adjudicated Claims is one of the largest US health plan claims databases and is comprised of adjudicated claims for more than 150 million unique enrollees across the US. The database is considered representative of the national, commercially insured population in terms of age and gender. Standard fields include inpatient and outpatient diagnoses and procedures, and retail and mail order prescription records and payments. All data are compliant with the Health Insurance Portability and Accountability Act (HIPAA) to protect patient’s privacy.

### Patient selection

US managed care enrollees were initially identified in IQVIA RWD Adjudicated Claims based on ≥1 medical claim with a diagnosis for CIDP (ICD-9-CM 357.81) between July 1, 2010 and June 30, 2014. Both confirmatory and non-confirmatory (i.e., ancillary) claims were considered. The date of the first claim was termed the “index date.” Patients were required to have confirmation of CIDP denoted by: 1) a subsequent confirmatory medical claim with a diagnosis for CIDP or 2) initiation of CIDP therapy within 1 year of the index date. CIDP therapy was identified based on a broad list of available therapies or procedures which have been proven or investigated in CIDP (Table 1) [3].

**Table 1:** CIDP therapies.

IVIg	Etanercept
Corticosteroids	Interferon B1a
Alemtuzumab	Methotrexate
Azathioprine	Mycophenolate mofetil
Cyclophosphamide	Natalizumab
Cyclosporine A	Rituximab
Plasma exchange	Tacrolimus
Hematopoietic stem cell transplantation	

Patients were required to have ≥6 months of continuous enrollment (CE) in the health plan before the index date (i.e., the 6-month baseline or pre-index period) and to have ≥2-years CE following the index date (i.e., the 2-year follow-up or post-index period). Patients were required to be newly diagnosed with CIDP as of the index date and were excluded if they had any medical claims with a diagnosis for CIDP or if they had any claims for CIDP therapy in the 6-month pre-index period. Finally, only adults were included (≥18 years old at index) and patients were excluded if they had poor data quality (i.e., incomplete or invalid data).

Patients with CIDP (cases) were matched to patients without CIDP (controls). Cases were direct matched to controls based on: age, gender, geographic region, health plan type, payer type and Charlson Comorbidity Index (CCI) score. The index date for controls was set as the index date of the matched patient with CIDP. Controls had neither medical claims with a diagnosis for CIDP nor claims for CIDP therapy at any time during the study period. Controls were required to be ≥18 years old, to have ≥6-months pre- and ≥2-years post-index CE, and to have adequate data quality.

### Study measures

Baseline demographic and clinical characteristics were assessed for cases and controls. Demographic characteristics included age, gender, health plan type, payer type and geographic region at index. Clinical characteristics were measured over the 6-month pre-index period and included the CCI, common and relevant comorbidities of interest, prior use of therapies of interest and total healthcare costs. Alternative pre-index diagnoses, i.e., occurring prior to confirmed CIDP diagnosis, which may be considered potential misdiagnoses [6] were also assessed.

Use of CIDP treatment was assessed over the 2-year follow-up among cases. Initiation of therapy was classified as either monotherapy or combination therapy based on the different therapies used within 30 days of therapy initiation (at the class level for corticosteroids or IVIg; at the generic product level for all other therapies). For instance, a patient with claims for two different IVIg products over the first 30 days of therapy initiation was classified as receiving IVIg monotherapy, while a patient with claims for a corticosteroid and an IVIg was classified as receiving combination therapy.

The clinical and economic burden of CIDP was assessed over the 2-year follow-up among cases and controls. Select clinical characteristics were also assessed over the 2-year follow-up, including new use of therapies of interest (without use in the pre-index). Healthcare resource use (HCRU) and costs were measured for cases and controls over the 2-year follow-up and compared to quantify the incremental burden of CIDP. Allowed healthcare costs, the amount paid by the health plan and patient combined, were evaluated on a per cohort member basis. Thus, the denominator included all patients in a cohort, regardless of whether they had utilization of a specific service. Costs were converted to 2016 US dollars (USD) using the medical component of the Consumer Price Index.

HCRU and costs were assessed for outpatient pharmacy and medical services. Medical services included the following mutually exclusive healthcare categories: hospitalizations and outpatient medical care (emergency room [ER] visits, physician office visits, outpatient surgical visits, laboratory and pathology, and outpatient ancillary, radiology and Healthcare Common Procedure Coding System [HCPCS] drugs). CIDP therapy costs (inclusive of both outpatient pharmacy and medical claims) were separately assessed.

### Statistical analyses

Descriptive analyses were organized as follow. For categorical measures, reporting included the frequency (number of patients [N]) and percentage (%) for each cohort. For continuous variables, both the mean (standard deviation [SD]) and median were reported. Study outcomes were compared between the matched cohorts using paired t-test (mean) and the Wilcoxon signed-rank test (median) for continuous variables and McNemar’s or Bowker’s test for categorical variables.

A generalized estimating equation model (GEE) was developed, with a gamma distribution and log-link function, to evaluate differences in total healthcare costs over the 2-year follow-up between matched cohorts. The model included baseline clinical characteristics measured over the 6-month pre-index period that were significantly different between matched cases and controls, including back pain, use of anti-depressants, anti-anxiety medications, anti-convulsants, benzodiazepines, central muscle relaxants, NSAIDs, and opioids, number of outpatient physician office visits and total healthcare costs.

A p-value of <0.05 was considered statistically significant. All analyses used SAS version 9.3 (SAS Institute Inc., Cary, NC).

## Results

### Patient sample

**Fig 1:**
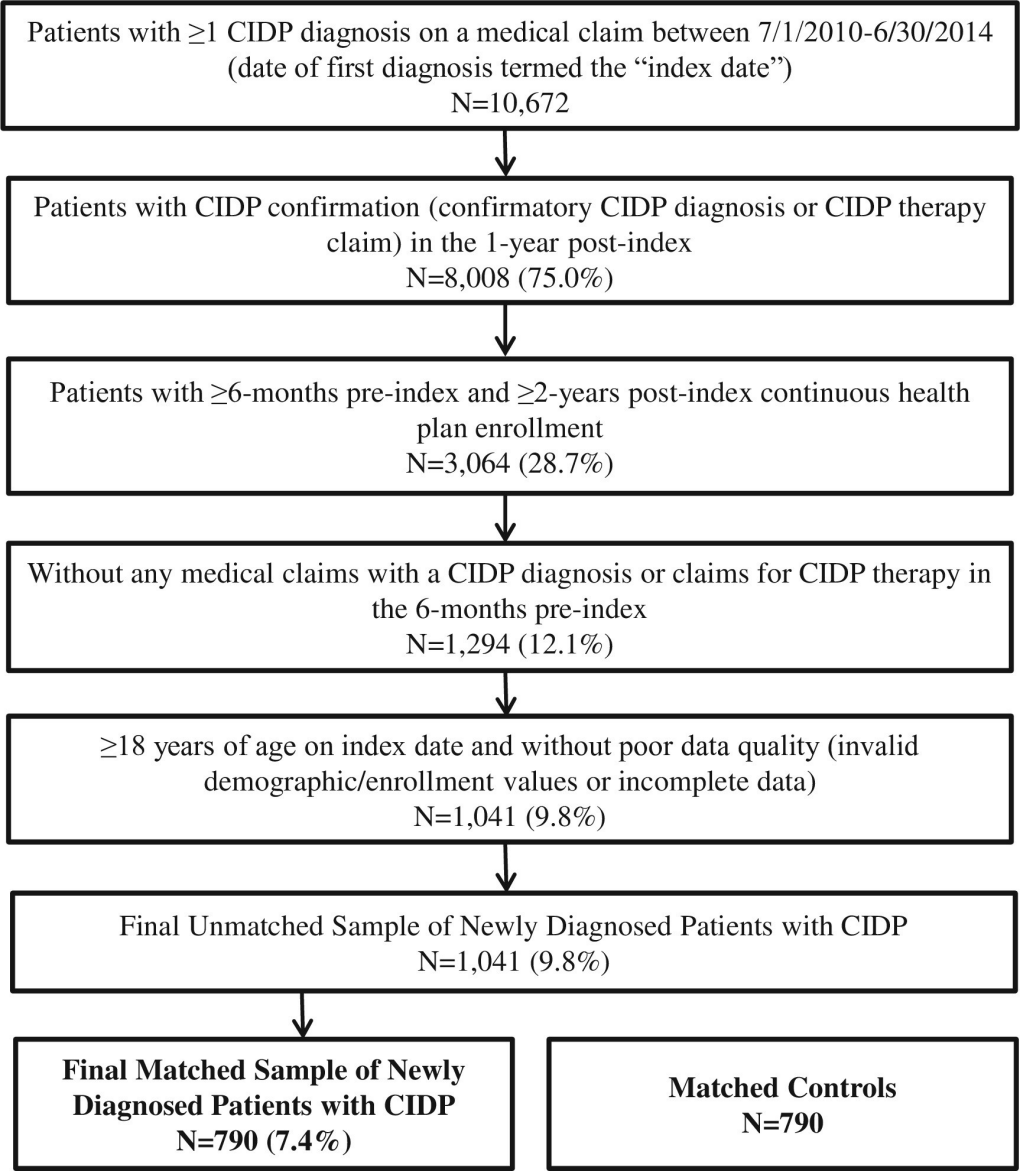
Study flow chart. CIDP = chronic inflammatory demyelinating polyneuropathy.

A total of 10,672 patients were identified with a medical claim with a diagnosis for CIDP between July 1, 2010 and June 30, 2014 and were evaluated for study inclusion (see Fig 1 for patient selection flow). Of the starting 10,672 patients, 8,008 (75.0%) had confirmation of CIDP within 1 year of the index date, while 3,064 (28.7%) met the CE requirements. The unmatched sample consisted of 1,041 (9.8%) patients with newly diagnosed CIDP. The final sample comprised 790 (7.4%) cases successfully matched to 790 controls without CIDP.

### Patient characteristics

Baseline demographic characteristics were direct matched between cases and controls (Table 2). Mean (SD) age at index for cases and controls was 49.7 (11.4). Half (53.7%) were male. Patients were most often located in the South (40.4%) and the majority was commercially-insured (63.9%).

**Table 2:** Baseline demographic characteristics and clinical characteristics in the 6-month pre-index period and 2-year follow-up.

	Baseline					2-Year Follow-Up				
	Cases N = 790		Controls N = 790		P value^b^	Cases N = 790		Controls N = 790		P value^b^
Characteristic	n	%	n	%		n	%	n	%	
Age (years)^a^										
Mean ± SD	49.7 ± 11.4		49.7 ± 11.4							
Median	52		52							
Male gender^a^	424	(53.7)	424	(53.7)						
Region^a^										
Northeast	219	(27.7)	219	(27.7)						
Midwest	167	(21.1)	167	(21.1)						
South	319	(40.4)	319	(40.4)						
West	85	(10.8)	85	(10.8)						
Payer type^a^										
Commercial	505	(63.9)	505	(63.9)						
Medicaid	19	(2.4)	19	(2.4)						
Medicare Risk	9	(1.1)	9	(1.1)						
Self-insured	257	(32.5)	257	(32.5)						
Health plan type^a^										
Consumer-directed	6	(0.8)	6	(0.8)						
HMO	80	(10.1)	80	(10.1)						
Indemnity	14	(1.8)	14	(1.8)						
POS	16	(2.0)	16	(2.0)						
PPO	674	(85.3)	674	(85.3)						
Index year^a^										
2010	136	(17.2)	136	(17.2)						
2011	226	(28.6)	226	(28.6)						
2012	176	(22.3)	176	(22.3)						
2013	175	(22.2)	175	(22.2)						
2014	77	(9.7)	77	(9.7)						
CCI^a^										
0	580	(73.4)	580	(73.4)		334	(42.3)	495	(62.7)	<.0001
1	120	(15.2)	120	(15.2)		154	(19.5)	138	(17.5)	0.2893
2	79	(10.0)	79	(10.0)		134	(17.0)	105	(13.3)	0.0388
3+	11	(1.4)	11	(1.4)		168	(21.3)	52	(6.6)	<.0001
Mean ± SD	0.4 ± 0.8		0.4 ± 0.8			1.5 ± 1.9		0.7 ± 1.3		<.0001
Median	0		0			1		0		<.0001
Common (≥5%) comorbidities of interest:										
Asthma/COPD	19	(2.4)	9	(1.1)	0.0588	65	(8.2)	17	(2.2)	<.0001
Back pain	241	(30.5)	80	(10.1)	<.0001	371	(47.0)	150	(19.0)	<.0001
Cardiac dysrhythmia	34	(4.3)	28	(3.5)	0.4386	100	(12.7)	50	(6.3)	<.0001
Cerebrovascular disease	25	(3.2)	12	(1.5)	0.0280	81	(10.3)	25	(3.2)	<.0001
CAD	22	(2.8)	23	(2.9)	0.8788	62	(7.8)	52	(6.6)	0.3124
Diabetes	87	(11.0)	112	(14.2)	0.0176	172	(21.8)	147	(18.6)	0.0867
Dyslipidemia	215	(27.2)	237	(30.0)	0.1806	361	(45.7)	363	(45.9)	0.9113
Hypertension	233	(29.5)	223	(28.2)	0.5543	364	(46.1)	314	(39.7)	0.0064
Hypothyroidism	80	(10.1)	53	(6.7)	0.0126	128	(16.2)	86	(10.9)	0.0016
IBD	16	(2.0)	14	(1.8)	0.7150	46	(5.8)	25	(3.2)	0.0103
Leukemia/lymphoma	26	(3.3)	6	(0.8)	0.0004	84	(10.6)	34	(4.3)	<.0001
Neuropathic pain	314	(39.7)	23	(2.9)	<.0001	455	(57.6)	54	(6.8)	<.0001
Osteoarthritis	65	(8.2)	26	(3.3)	<.0001	188	(23.8)	62	(7.8)	<.0001
PVD	17	(2.2)	3	(0.4)	0.0017	50	(6.3)	13	(1.6)	<.0001
Sleep apnea	14	(1.8)	5	(0.6)	0.0290	59	(7.5)	23	(2.9)	<.0001
Common (≥5%) therapies of interest:										
Anti-anxiety medications	14	(1.8)	5	(0.6)	0.0389	50	(6.3)	17	(2.2)	<.0001
Anti-convulsants	242	(30.6)	40	(5.1)	<.0001	361	(45.7)	57	(7.2)	<.0001
Anti-depressants	216	(27.3)	108	(13.7)	<.0001	349	(44.2)	144	(18.2)	<.0001
Benzodiazepines	121	(15.3)	43	(5.4)	<.0001	207	(26.2)	75	(9.5)	<.0001
Central muscle relaxants	93	(11.8)	42	(5.3)	<.0001	206	(26.1)	71	(9.0)	<.0001
Lidocaine	18	(2.3)	0	(0.0)	-	52	(6.6)	10	(1.3)	<.0001
NSAIDs	142	(18.0)	71	(9.0)	<.0001	253	(32.0)	137	(17.3)	<.0001
Opioids	264	(33.4)	128	(16.2)	<.0001	479	(60.6)	212	(26.8)	<.0001

^a^Cases (N = 790) and Controls (N = 790) were direct matched (exact matched) on demographic variables and CCI.

^b^McNemar’s or Bowker’s test for categorical variables, and paired t-test (mean) and the Wilcoxon signed-rank test (median) for continuous variables.

CAD = Coronary artery disease; CCI = Charlson Comorbidity Index; COPD = chronic obstructive pulmonary disease; HMO = health maintenance organization; IBD = Inflammatory bowel disease; NSAIDs = nonsteroidal anti-inflammatory drugs; PAD = Peripheral vascular disease; POS = point of service; PPO = preferred provider organization; SD = standard deviation.

Baseline clinical characteristics can be found in Table 2. Patients were also direct matched on CCI score and the majority (73.4%) had a CCI score of 0 over the 6-month pre-index period. Among cases, alternative, pre-index diagnoses that may be considered as misdiagnosis of CIDP included multifocal motor neuropathy (31.1%), fibromyalgia (9.9%), Guillain-Barré syndrome (5.8%), multiple sclerosis (5.6%) and idiopathic small fiber neuropathy (4.7%). Overall, 47.7% of cases had a *prior* alternative diagnosis compared to 3.3% of controls (p<0.0001). A few comorbidities in the 6-month pre-index period were significantly higher among cases compared to controls including neuropathic pain (39.7% vs. 2.9%), back pain (30.5% vs. 10.1%) and osteoarthritis (8.2% vs. 3.3%) (all p<0.0001).

A number of therapies of interest were more frequently used in the 6-month pre-index period among cases compared to controls including anti-convulsants (30.6% vs. 5.1%), opioids (33.4% vs. 16.2%) and anti-depressants (27.3% vs. 13.7%) (all p<0.0001). More cases had a hospitalization compared to controls (6.7% vs. 3.8%, p = 0.0056). Cases also had a higher mean number of outpatient physician office visits (8.0 vs. 4.1) and higher mean total costs ($8,316 vs. $3,748) (both p<0.0001). In the 6-month pre-index period, 39.4% of cases had ≥1 physician office visit to a neurologist and 10.4% had ≥1 physician office visit to an orthopedic surgeon, compared to 2.2% and 3.5% of controls, respectively.

### Treatment utilization over the 2-year follow-up

Over the 2-year follow-up, 657 patients (83.2%) initiated CIDP therapy in a median of 52 days. Among patients that initiated CIDP therapy, the majority (57.4%) initiated monotherapy with corticosteroids, while 27.5% initiated monotherapy with IVIg and 8.2% initiated combination therapy with corticosteroids and IVIg. Among patients that initiated CIDP therapy, 83.1% had any use of corticosteroids and 41.2% had any use of IVIg over the entire 2-year follow-up; utilization of other CIDP treatments was infrequent (<5%) and included mycophenolate mofetil (4.4%), azathioprine (4.1%) and PE (4.0%).

### Clinical burden over the 2-year follow-up

Over the 2-year follow-up, CCI score was higher for cases than controls (median 1 vs. 0, p<0.0001) (Table 2). A number of comorbidities of interest were significantly higher among cases compared to controls including neuropathic pain (57.6% vs. 6.8%), back pain (47.0% vs. 19.0%) and osteoarthritis (23.8% vs. 7.8%) (all p<0.0001). Several therapies of interest were more frequently used among cases compared to controls including opioids (60.6% vs. 26.8%), anti-convulsants (45.7% vs. 7.2%) and anti-depressants (44.2% vs. 18.2%) (all p<0.0001). More cases were new to therapies of interest (which were not observed in the pre-index) than controls, including opioids (31.4% vs. 19.5%, p<0.0001), NSAIDs (20.8% vs. 13.5%, p = 0.0002), anti-depressants (19.9% vs. 6.1%, p<0.0001) and anti-convulsants (19.2% vs. 2.8%, p<0.0001).

### Economic burden over the 2-year follow-up

**Fig 2:**
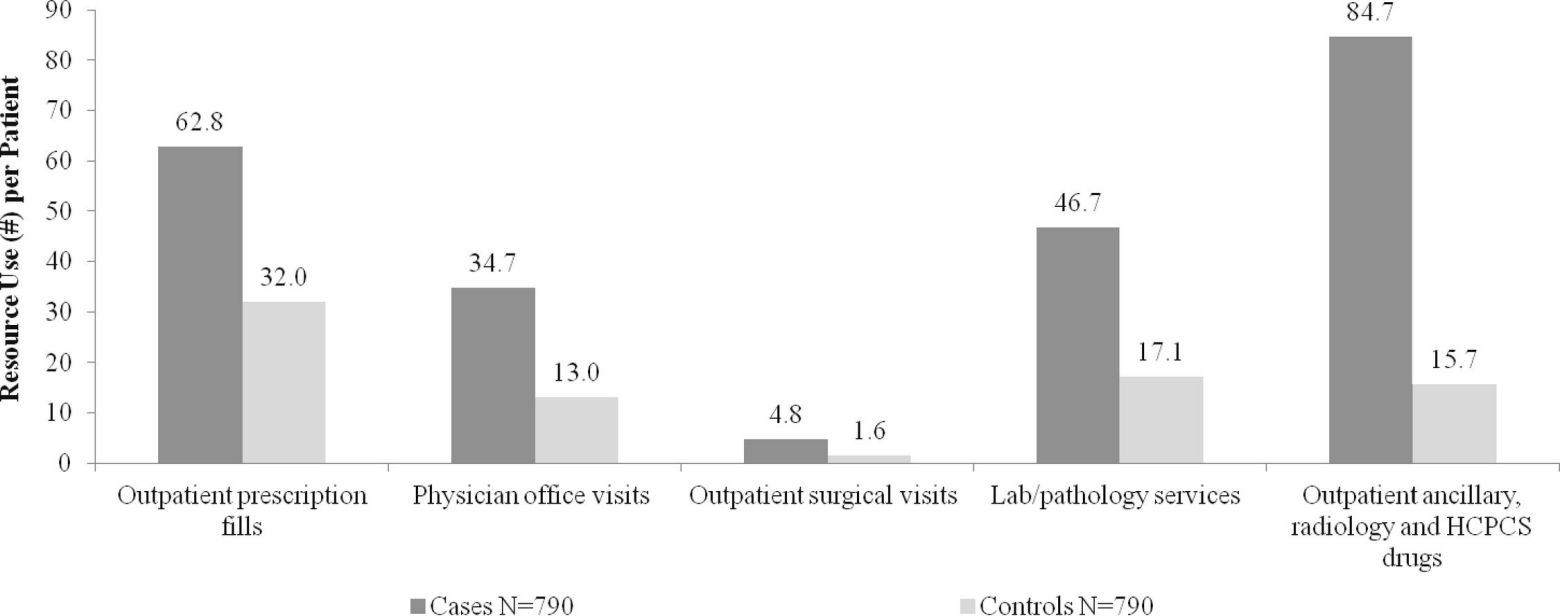
Mean resource use (#) per patient over the 2-year follow-up. All p<0.0001. ER = emergency room; HCPCS = Healthcare Common Procedure Coding System.

Over the 2-year follow-up, HCRU was significantly higher among cases. Proportion with utilization of a service was significantly higher among cases compared to controls including proportion with ≥1 hospitalization (26.2% vs. 9.0%), ER visit (42.2% vs. 21.9%) or outpatient surgical visit (77.1% vs. 45.9%) (all p<0.0001). More cases had ≥1 neurologist office visit (67.0% vs. 3.9%) or physical therapy visit (14.7% vs. 2.8%) (both p<0.0001). Number of healthcare services per patient was also significantly higher among cases including mean number of outpatient prescription fills (62.8 vs. 32.0) and physician office visits (34.7 vs. 13.0) (both p<0.0001) (Fig 2).

**Fig 3:**
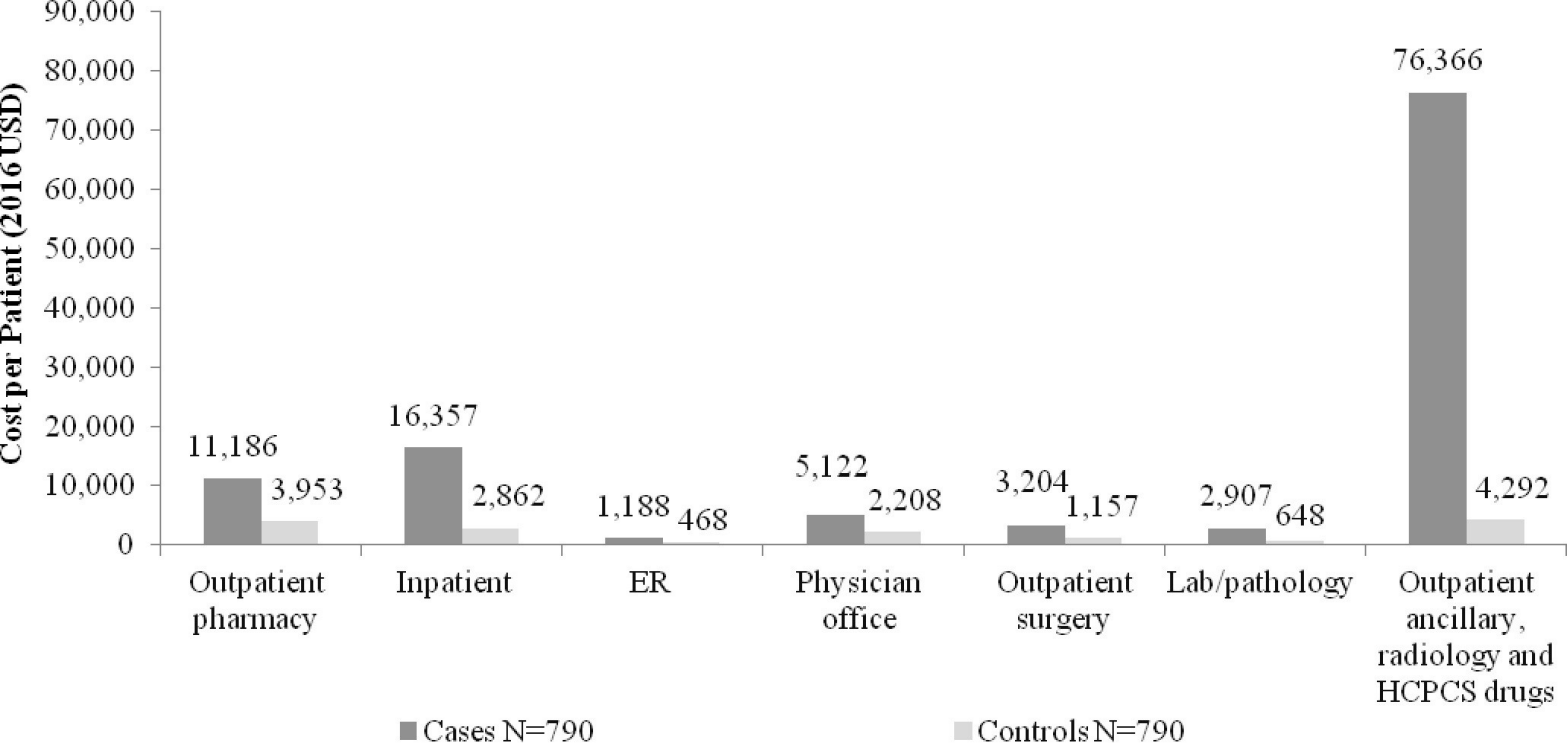
Mean healthcare cost per patient over the 2-year follow-up. All p<0.0001 except for ER (p = 0.0003). ER = emergency room; HCPCS = Healthcare Common Procedure Coding System; USD = US dollar.

Over the 2-year follow-up, total costs were significantly higher for cases than controls (mean $116,330 vs. $15,586, p<0.0001) (See Table 3 and Fig 3). Compared to controls, cases had 7.5x higher mean total costs. Healthcare costs were significantly higher among cases for all resource categories, including mean per patient cost for outpatient ancillary, radiology and HCPCS drugs ($76,366 vs. $4,292), inpatient care ($16,357 vs. $2,862) and physician office visits ($5,122 vs. $2,208) (all p<0.0001). Compared to controls, cases had 17.8x higher costs for outpatient ancillary, radiology and HCPCS drugs, 9.0x higher medical costs and 5.7x higher inpatient costs. As a proportion of mean total costs for cases and controls, medical costs represented 90.4% and 74.6%, costs for outpatient ancillary, radiology and HCPCS drugs represented 65.6% and 27.5%, and inpatient costs represented 14.1% and 18.4%, respectively. Among cases, mean (SD) cost of CIDP therapy per patient (inclusive of outpatient pharmacy and medical claims) was $59,619 ($136,892), and CIDP therapy accounted for 51.2% of total costs. Note that 17% of cases used no CIDP therapy over the 2-year follow-up, contributing to the observed high standard deviation and wider distribution of costs.

**Table 3:** Healthcare cost per patient over the 2-year follow-up.

	Cases			Controls			P value*	P value*
	N = 790			N = 790				
Cost (2016 USD) per patient	Mean	SD	Median	Mean	SD	Median	Mean	Median
**Outpatient Pharmacy**	11,186	24,947	3,014	3,953	10,574	848	<.0001	<.0001
**Medical**	105,144	177,800	33,206	11,633	55,090	3,144	<.0001	<.0001
Inpatient	16,357	66,522	0	2,862	17,257	0	<.0001	<.0001
ER	1,188	5,168	0	468	2,215	0	<.0001	0.0003
Physician office	5,122	10,297	3,015	2,208	20,115	943	<.0001	0.0003
Outpatient surgery	3,204	8,172	809	1,157	4,372	0	<.0001	<.0001
Lab/pathology	2,907	11,012	1,120	648	1,903	278	<.0001	<.0001
Outpatient ancillary, radiology and HCPCS drugs	76,366	155,863	12,355	4,292	23,125	800	<.0001	<.0001
**TOTAL COST**	**116,330**	**179,116**	**47,827**	**15,586**	**56,692**	**5,823**	**<.0001**	**<.0001**
**Total CIDP Therapy**	59,619	136,892	59					

*Paired t-test (mean) and the Wilcoxon signed-rank test (median).

CIDP = chronic inflammatory demyelinating polyneuropathy; ER = emergency room; HCPCS = Healthcare Common Procedure Coding System; USD = US dollar.

In the GEE of total healthcare costs over the 2-year follow-up, cases were associated with a 6.1x increase in total costs compared to controls (p<0.0001).

## Discussion

This study represents to our knowledge the first real-world database study to quantify the incremental burden of CIDP using a matched case-control analysis. Our findings suggest a substantial clinical and economic burden among patients with CIDP compared to matched controls. Over the 2-year follow-up, common symptoms of CIDP included neuropathic pain, back pain and osteoarthritis. Medications such as anti-convulsants, anti-depressants and even opioids were frequently utilized. A third of cases newly used opioids following initial diagnosis. We observed that a number of clinical characteristics appeared to increase for cases in the 2-year follow-up compared to the 6-month pre-index including CCI score, and presence of neuropathic pain, back pain and osteoarthritis. Over the 2-year follow-up, cases had significantly higher HCRU compared to controls, including more patients with ≥1 hospitalization and a higher number of physician office visits. Cases were associated with 7.5x higher mean total healthcare costs compared to controls. Outpatient ancillary, radiology and HCPCS drugs comprised the majority of total costs, while inpatient care was also an important cost driver. Half of total costs for cases over the 2-year follow-up were attributable to CIDP therapy (outpatient prescriptions, HCPCS drugs and therapeutic procedures). Following further adjustment in the GEE, cases were associated with a 6-fold increase in total healthcare costs compared to controls.

The frequent use of opioids observed among CIDP cases is concerning. We have no way of identifying whether opioids were prescribed for the CIDP or, as is likely, for observed comorbid neuropathic or chronic pain due to the limitations of the study database. While severe pain is unusual in CIDP, it appears that comorbid pain-related conditions were relatively common among our cases. We found that almost half of cases experienced back pain and almost a quarter experienced osteoarthritis over the follow-up. Yet, the use of opioids, even if for neuropathic pain, remains controversial and opioids do not provide improvements in physical functioning among those with neuropathic pain [14,15]. Further, opioids should not be used as first-line or routine therapy for chronic pain, and their use must be carefully considered given the potential for misuse, abuse and overdose [16].

Only one identified study has previously evaluated total healthcare costs among CIDP patients in the US [12]. A total of 73 patients with CIDP were identified from 9 small commercial health plans. The mean health plan paid cost per patient in 2011 was $56,953. A quarter (26%) of patients had claims for IVIg, while 16% had claims for prednisone. This study highlighted the high costs of IVIg: 90% of pharmacy costs were related to IVIg, which was administered to only a minority (26%). Our study, conducted using a large, nationally representative database and with a much larger sample size, found a mean 2-year total cost of $116,330, consistent with being approximately double the annual cost found by Guptill et al. An older study quantified the cost-of-illness of CIDP in 2008 in southeast England [13]. The total annual cost-of-illness per patient was £22,085 for CIDP. The use of IVIg was the most important determinant of cost.

We did not evaluate costs of specific CIDP therapies in this analysis. In an exploratory analysis, we observed that patients initiating monotherapy with IVIg had higher CIDP therapy costs than patients initiating monotherapy with corticosteroids. From treatment initiation to the end of the 24-month follow-up, corticosteroid patients had mean (SD) CIDP therapy costs of about $7,900 ($35,000) while IVIg patients had mean CIDP therapy costs of $165,000 ($170,000). IVIg patients had higher costs, in part, related to longer persistence on IVIg compared to corticosteroids. The long-term clinical and economic impact of greater discontinuation of steroids compared to IVIg is unknown. Further, the claims data do not provide any insights into clinical effectiveness. Yet, our observation of high CIDP therapy costs, and high costs of IVIg specifically, highlights the importance of optimally managing the treatment of CIDP while considering both clinical benefit and costs. Setting of care has also been identified as an important cost driver for IVIg, with IVIg administration in the outpatient hospital setting associated with higher costs compared to IVIg administration in the home setting [17,18]. While we did not specifically evaluate IVIg costs, setting of care is not comprehensively recorded in the database for IVIg administration, limiting our ability to investigate this cost driver.

This study has limitations inherent to retrospective database studies, as well as to the data source and study design. Results from retrospective studies should be interpreted with understanding of their inherent limitations, and in context of results from other similar studies. Administrative databases do not provide as much clinical detail, for example, treatment outcomes or disease severity, as medical records. We relied on diagnosis and therapy codes on administrative billing claims to identify patients with CIDP; however, CIDP is often misdiagnosed [6,7] Although we required an additional CIDP diagnosis claim and/or initiation of CIDP-specific treatment to confirm the CIDP diagnosis, in the absence of clinical data (electronic medical records, chart review, etc.) to confirm CIDP, some diagnostic uncertainty still remains. There is limited visibility into healthcare resource use or prescriptions obtained outside of the plan benefit. However, 70+ plans contribute to the database and any unobserved out-of-network claims are likely to be very limited. Our study only captures the direct cost of CIDP as measured by administrative claims data. The data provide no insight into indirect costs of CIDP such as loss of productivity or unemployment, or insight into the quality of life impacts of CIDP, such as functional limitations, fatigue, pain, anxiety and depression [19]. Thus, the comprehensive societal impact of CIDP remains unknown. Continuous health plan enrollment was required for inclusion in the study to eliminate the impact of insurance coverage interruptions and to have full visibility into healthcare resource utilization and associated costs obtained through the plan benefit. This requirement may bias the analysis towards a healthier sample (by excluding patients who disenrolled due to death, changes in employment, or for other reasons during this period). And because CIDP is a chronic condition, it is possible that establishment of a treatment schedule could take several years for some patients. Thus, the economic burden of CIDP quantified in this study may represent a conservative estimate. Finally, since the study sample employed was largely commercially- or self-insured, these findings are not readily generalizable to uninsured, Medicare or Medicaid populations. Despite these limitations, our study is the first to report estimates of the healthcare resource utilization and costs associated with CIDP at a national level in the US, as well as the incremental burden of CIDP.

Our study constitutes the largest retrospective real-world database study of the economic burden of CIDP in the US and is the only study to quantify the incremental burden of CIDP relative to matched controls. Our findings suggest a substantial clinical and economic burden associated with CIDP. Over the 2-year follow-up, cases had higher healthcare resource utilization which was associated with 7.5x higher mean total healthcare costs compared to controls. This study provides important insights into the economic burden of CIDP; however, future studies are necessary to understand the economic burden among different patient populations as well as from indirect and societal perspectives. Future research is also needed to investigate optimal therapeutic strategies in CIDP to balance the costs and outcomes of therapy.
